# Clinical presentation of asymptomatic and symptomatic heterosexual men who tested positive for urethral gonorrhoea at a sexual health clinic in Melbourne, Australia

**DOI:** 10.1186/s12879-020-05197-y

**Published:** 2020-07-08

**Authors:** Mario Martín-Sánchez, Jason J. Ong, Christopher K. Fairley, Marcus Y. Chen, Deborah A. Williamson, Kate Maddaford, Ei T. Aung, Georgia Carter, Catriona S. Bradshaw, Eric P. F. Chow

**Affiliations:** 1 Melbourne Sexual Health Centre, Alfred Health, 580 Swanston Street, Carlton, Melbourne, VIC 3053 Australia; 2 Preventive Medicine and Public Health Training Unit PSMar-UPF-ASPB, Barcelona, Spain; 3 Public Health Agency of Barcelona, Plaça de Lesseps, 1, 08023 Barcelona, Spain; 4 Central Clinical School, Monash University, Melbourne, VIC Australia; 5 Microbiological Diagnostic Unit Public Health Laboratory, Department of Microbiology and Immunology, The University of Melbourne at The Peter Doherty Institute for Infection and Immunity, Melbourne, Australia; 6 Centre for Epidemiology and Biostatistics, Melbourne School of Population and Global Health, The University of Melbourne, Carlton, Victoria Australia

**Keywords:** Sexually transmitted infections, Screening, Testing, Urogenital, Genital, Discharge, Men who have sex with women, MSM, *Neisseria gonorrhoeae*

## Abstract

**Background:**

Asymptomatic screening for gonorrhoea in heterosexual men is currently not recommended in many countries including Australia, given the prevalence is relatively low in the heterosexual population. We aimed to determine the proportion of urethral gonorrhoea cases among heterosexual men attending a sexual health clinic that was asymptomatic and symptomatic, the time since last sexual contact to the onset of symptoms and the time to clinic presentation following the onset of symptoms.

**Methods:**

This was a cross-sectional study that included heterosexual men aged 16 years or above attending the Melbourne Sexual Health Centre (MSHC) in Australia between August 2017 and August 2018. Gonorrhoea cases were diagnosed by nucleic acid amplification testing (NAAT) and/or culture. Descriptive analyses were conducted for all gonorrhoea cases including demographic characteristics, recent sexual practices, reported urethral symptoms and duration, sexual contact with a person diagnosed with gonorrhoea, investigations performed and laboratory results.

**Results:**

There were 116 confirmed cases of urethral gonorrhoea in heterosexual men over the study period of which 6.0% (95% CI: 2.7–12.1%) were asymptomatic. Typical urethral discharge was present in 80.2% (95% CI: 71.9–86.5%) of men. The mean time between last sexual contact and the onset of symptoms was 7.0 days, and between the onset of symptoms to presentation to the clinic was 5.6 days.

**Conclusions:**

A small proportion of heterosexual men with urethral gonorrhoea do not have any symptoms. Heterosexual men with urethral symptoms usually seek for healthcare within a week, prompting rapid healthcare-seeking behaviour.

## Background

In Australia, gonorrhoea cases among both men (from 91.1 to 174.2 notifications per 100,000 population) and women (from 39.6 to 61.8 notifications per 100,000 population) have increased between 2013 and 2017 [1]. Similar rises are reported in other countries such as the US and UK [2]. The increase in gonorrhoea cases had until recently been occurring primarily in gay, bisexual and other men who have sex with men (MSM) [3–6].

The reasons for the increase in cases of gonorrhoea in heterosexuals are not clear. The Australian Study of Health and Relationships surveys reported that there was no change in condom use, or in the number of sexual partners among heterosexuals between 2001 and 2013 [7, 8]. However, the increase in health care demand resulting from the rising rates of sexually transmitted infections (STIs) may be making it harder to access health care in a timely manner, increasing the duration of symptomatic infection, which would increase the reproductive rate of infection [9–11]. An alternative explanation is that there is “bridging” from rising rates in MSM to the heterosexual population. This explanation is supported by a study that undertook whole genome sequencing (WGS) of *Neisseria gonorrhoeae* isolates from Victoria, Australia [12], which showed distinct *N. gonorrhoeae* lineages circulating that included both MSM and heterosexuals, indicating bridging of strains between these subpopulations.

Ong and colleagues addressed this latter explanation in MSM attending a sexual health clinic in Melbourne between 2015 and 2016, and estimated that 11% of MSM diagnosed with urethral gonorrhoea by nucleic acid amplification test (NAAT) did not have any urethral symptoms at the time of diagnosis. Among symptomatic individuals with typical urethral discharge for gonorrhoea, the average time since their last sexual act was 3.9 days and the mean time between the onset of symptoms to presentation to the clinic was 3 days. Among symptomatic individuals with atypical urethral symptoms for gonorrhoea, the average time since their last sexual act was 6 days and the mean time between the onset of symptoms to the presentation to the clinic was 2 days [13].

The primary aim of this study was to determine the proportion of urethral gonorrhoea among heterosexual men attending a sexual health clinic that was asymptomatic or symptomatic detected by NAAT. The secondary aim was to estimate the time since last sexual contact to the onset of symptoms and the time to healthcare seeking following the onset of symptoms among heterosexual men with urethral gonorrhoea.

## Methods

### Study population

This was a cross-sectional study including heterosexual men aged 16 years or above attending the Melbourne Sexual Health Centre (MSHC) in Australia between August 2017 and August 2018. For this study, we defined heterosexual men as men who have sex with women only in the preceding 12 months.

Prior to August 2017, urethral gonorrhoea testing at MSHC was only performed among heterosexual men who reported genital symptoms or contact of infection. However, our clinic changed its testing policy and guidelines in August 2017, where men attending MSHC were offered for both urethral gonorrhoea and chlamydia testing, regardless of the presence of symptoms or the sex of their partners. All men attending MSHC were asked if they had had symptoms by the triage nurse and again by the treating clinician. Men with no urethral symptoms were screened for urethral gonorrhoea by NAAT in first-pass urine. Heterosexual men who reported urethral discharge or other relevant urogenital symptoms (e.g. dysuria, urethral discomfort) underwent genital examination and had a urethral swab collected for NAAT and/or culture, in addition to the urine sample. The urine samples were tested by nucleic acid amplification test (NAAT) using Aptima Combo 2 (AC2) assay (Hologic Panther system; Hologic, San Diego, CA, USA). If a urethral swab was collected for culture, it was immediately plated onto modified Thayer-Martin medium for gonorrhoea culture and a smear prepared for Gram stain and microscopy to look for Gram-negative diplococci. For men who tested positive by NAAT but had no culture performed on the day of screening, a culture for gonorrhoea was performed on the day when they returned for treatment, but before the treatment was administered, to determine the antimicrobial susceptibility profile.

### Data collection

We collected data regarding demographic characteristics, recent sexual practices, reported urethral symptoms and duration of symptoms, sexual contact with a person diagnosed with gonorrhoea, clinical investigations performed and laboratory results. For this study, we categorised men into three groups based on their reported symptoms: (1) typical discharge; (2) atypical symptoms; or (3) no symptoms. Typical urethral discharge was defined as purulent discharge, that is, yellow, green or pus-like discharge present on the day of testing. Atypical symptoms were urethral symptoms suggestive of urethritis other than purulent discharge, namely, dysuria, urethral discomfort, or non-purulent discharge. “No symptoms” was defined as individuals who reported no urethral symptoms on the day of testing.

### Data analysis

Descriptive analyses were conducted to summarize the key parameters. The 95% confidence intervals (CIs) for the sample proportions were calculated using Agresti-Coull (adjusted Wald) method. All analyses were conducted using SPSS version 25 (IBM, Chicago, USA). Ethical approval was granted by the Alfred Hospital Ethics Committee, Melbourne, Australia (450/18).

## Results

**Fig. 1:**
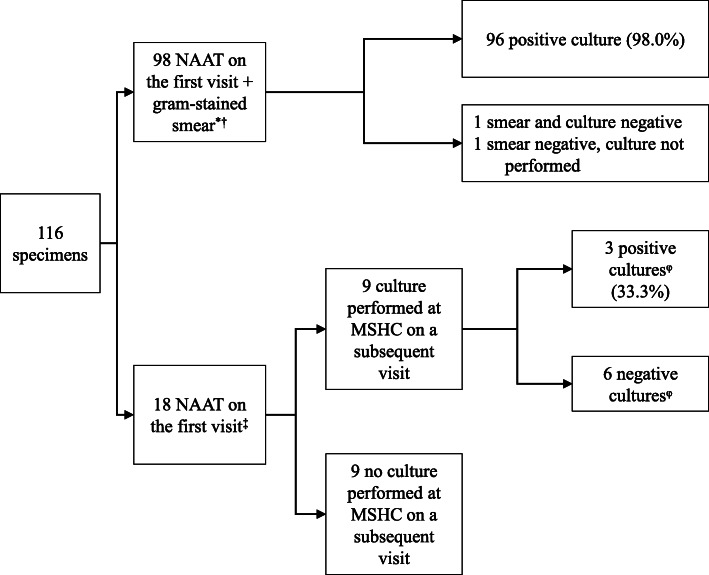
Investigations performed in 116 urethral *Neisseria gonorrhoeae* infections among heterosexual men. NAAT, nucleic acid amplification test; MSHC, Melbourne Sexual Health Centre. *92 (93.9%) had typical discharge and six other atypical symptoms (6.1%). † In one specimen, only culture was performed as it was previously diagnosed outside MSHC. ‡10 (55.6%) had atypical symptoms, seven had no symptoms (38.9%) and one typical discharge (5.6%). ^φ^Among the six consultations with a negative culture, five had previously received treatment for non-gonococcal urethritis (doxycycline 100 mg twice daily for 7 days) on the first visit, while none of the positive cultures had received treatment

There were 13,415 clinic visits by 8309 individual heterosexual men attending MSHC during the study period. The reasons for attendance to the clinic could be multiple for each individual man, the most frequent reasons were asymptomatic screening (*n* = 4543; 33.9%), presence of symptoms (*n* = 3587; 26.7%) and contact with an infected person (*n* = 723; 5.4%). There were 8686 consultations by 6388 individual men in which urethral gonorrhoea testing was performed. Consultations where men did not test for urethral gonorrhoea were mainly due to the return for a clinical review or treatment. Among the consultations in which the test was performed, urethral gonorrhoea positivity was 1.3% (116/8686), corresponding to 116 different gonorrhoea infections in 108 individual men (Fig. 1). There were eight men who tested positive twice for urethral gonorrhoea during the study period and were considered to have two different infections, given the clinical resolution of symptoms of the first episode and the time elapsed between the two positive results.

Among the 116 cases of urethral gonorrhoea, the median age was 31 (interquartile range 26–40), 55 (47.4%) were born overseas, three (2.6%) men were living with HIV and 14 (11.8%) had a prior history of a resolved *N. gonorrhoeae* infection at MSHC. The median number of female sexual partners in the preceding 3 months was three (interquartile range 2–4). Eighty-seven cases (75.0%) reported condomless sex with females in the preceding 3 months, 39 cases (33.6%) reported sex overseas and 16 (13.8%) reported sex with a sex worker in the preceding 12 months (Table 1).

**Table 1:** Demographic and sexual practices among 116 heterosexual men with urethral *Neisseria gonorrhoeae* infections attending Melbourne Sexual Health Centre, 2017–2018

Characteristics	***n*** (%)
Age (years)	
16–25	27 (23.3)
26–35	51 (44.0)
≥ 36	38 (32.8)
Country of birth	
Australia	56 (48.3)
Overseas	55 (47.4)
No information	5 (4.3)
Indigenous status	
Indigenous origin	0 (0)
Non-Indigenous origin	96 (82.8)
No information	20 (17.2)
Number of female sexual partners in the preceding 3 months, median [IQR]^a^	3 [2–4]
Number of female sexual partners in the preceding 3 months	
≤ 4	73 (62.9)
> 4	34 (29.3)
No information	9 (7.8)
Condom use with female sexual partners in the preceding 3 months^b^	
Always	16 (13.8)
Not always	87 (75.0)
No information	13 (11.2)
Sexual contact with a woman diagnosed with gonorrhoea	
Yes	9 (7.8)
No	107 (92.2)
Sex with sex worker in the last 12 months	
Yes	16 (13.8)
No	61 (52.6)
No information	39 (33.6)
Sex overseas in the last 12 months	
Yes	39 (33.6)
No	68 (58.6)
No information	9 (7.8)

*IQR* interquartile range, *NA* not applicable

^a^ The median was calculated among 107 heterosexual men

^b^ ‘Not always’ was defined as men who sometimes, usually or never used a condom with their female partners in the preceding 3 months

There were 93 (80.2% [95% CI: 71.9-86.5%]) cases who self-reported typical urethral discharge, 16 (13.8% [95% CI: 8.6-21.3%]) cases who self-reported atypical urethral symptoms and seven (6.0% [95% CI: 2.7-12.1%]) cases who self-reported no symptoms at the time of diagnosis (Table 2). Characteristics of the seven asymptomatic cases are listed in Table 3. One of these seven cases had been investigated for urethral symptoms 3 months before the diagnosis, one other had an ocular *N. gonorrhoeae* infection and another reported symptoms by the time when he returned for treatment. Of nine (7.8%) cases who reported sexual contact with a female diagnosed with gonorrhoea: four reported typical discharge, three did not have any symptoms and two reported other atypical symptoms.

**Table 2:** Clinical characteristics of urethral *Neisseria gonorrhoeae* infections among heterosexual men attending Melbourne Sexual Health Centre, 2017–2018

Characteristics	***n/N***	Percent (95% CI)
**History**		
Urethral symptoms reported on the day of the test		
Typical discharge	93/116	80.2 (71.9–86.5)
Atypical symptoms	16/116	13.8 (8.6–21.3)
No symptoms	7/116	6.0 (2.7–12.1)
**Investigations**^**a**^		
Gonorrhoea culture positive from the urethral swab		
Typical discharge	90/91	98.9 (93.4–100)
Atypical symptoms	6/12	50 (25.4–74.6)
No symptoms	3/3	100 (47.0–100)
Urine chlamydia positive		
Typical discharge	20/89	22.5 (15.0–32.3)
Atypical symptoms	5/16	31.3 (13.9–55.9)
No symptoms	2/7	28.6 (7.6–64.8)

^a^ The denominator of the percentage is the total of specimens in which the investigation was performed. *CI* confidence interval, *NAAT* nucleic acid amplification test

**Table 3:** Characteristics of the seven asymptomatic urethral *Neisseria gonorrhoeae* infection cases attending Melbourne Sexual Health Centre, 2017–2018

ID	Age group	Country of birth	Number of days between last sexual contact and clinic presentation	Number of sexual partners in the preceding 3 months	Condom use in the preceding 3 months	Sex with sex worker in the preceding 12 months	Sex overseas in the preceding 12 months, country	Previous gonorrhoea infection	Known gonorrhoea contact	Diagnostic pathway	Urethral symptoms	Non-urethral symptoms	Urethral chlamydia infection
1	20–24	Australia	Unknown	5	Sometimes	Unknown	Yes, Germany	No	No	NAAT on the first visit, positive culture in a subsequent visit	No	No	No
2	35–39	Australia	21	1	Sometimes	No	No	Yes	Yes	NAAT, no culture performed	No	No	No
3	60–64	Australia	Unknown	1	Sometimes	No	No	No	No	NAAT, no urethral culture performed, positive eye culture	No	Eye with purulent discharge	No
4	15–19	Australia	Unknown	25	Sometimes	Unknown	Unknown	No	No	NAAT on the first visit, positive culture in a subsequent visit	No	No	No
5	20–24	Australia	14	3	Always	Unknown	No	No	Yes	NAAT, no culture performed	Intermittent dysuria, testicular pain and discharge 3 months ago	No	Yes
6	40–44	Australia	7	3	Sometimes	Unknown	No	No	Yes	NAAT, no culture performed	No	No	No
7	25–29	England	14	3	Sometimes	No	No	No	No	NAAT on the first visit, positive culture in a subsequent visit	Not on the first visit, discharge in the subsequent visit	No	Yes

*NAAT* nucleic acid amplification test

**Fig. 2:**
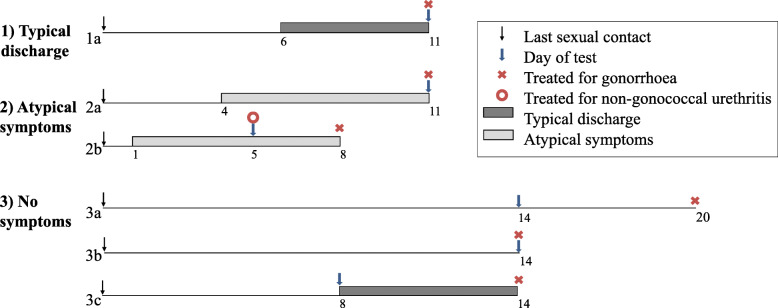
Mean interval (days) between the last reported sexual contact with women, the onset of urethral symptoms, testing and treatment in urethral *Neisseria gonorrhoeae* infections among heterosexual men stratified by symptom category. **(1a)** 93 (80.2%) men with typical discharge treated for gonorrhoea (azithromycin 1g plus ceftriaxone 500 mg) on the day of screening, 72 of them had time interval data reported. **(2a)** 9 (7.8%) men with atypical symptoms treated for gonorrhoea on the day of screening, 6 had time interval data reported; **(2b)** 7 (6.0%) men with atypical symptoms treated for non-gonococcal urethritis (azithromycin 1g or doxycycline 100 mg twice daily for 7 days) on the day of screening, 3 had time interval data reported. **(3a)** 4 (3.5%) men with no symptoms not treated for gonorrhoea on the day of screening, 2 had time interval data reported; **(3b)** 2 (1.7%) men with no symptoms treated for gonorrhoea on the day of screening, 1 had time interval data reported; **(3c)** 1 (0.9%) men with no symptoms on the day of screening but developed typical discharge later

**Fig. 3:**
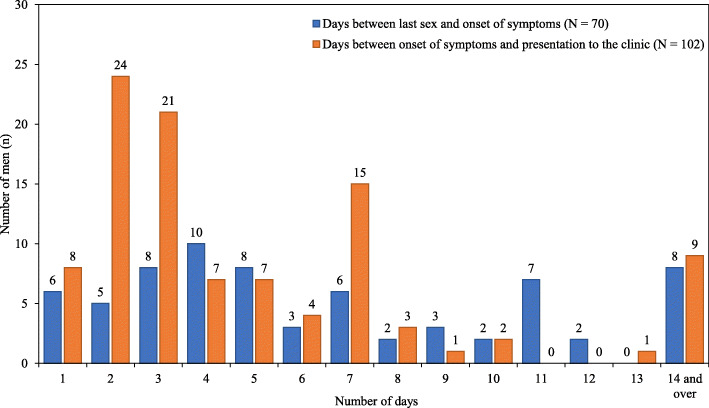
Mean interval (days) between the last sexual contact with women and the onset of urethral symptoms; and between the onset of urethral symptoms and presentation to the clinic, among heterosexual men diagnosed with urethral *Neisseria gonorrhoeae*. Note: There were 70 men who provided validated data on the day between last sex and onset of symptoms and 102 who men provided data on the day between onset of symptoms and presentation to the clinic

Figure 2 shows the mean time interval between reported last sexual contact, the onset of urethral symptoms if reported, testing and treatment for gonorrhoea stratified by symptom category. Among cases with symptoms, the mean time between last sexual contact and onset of symptoms was 7.0 days (standard deviation [SD] 5.4, ranging from 1 to 33 days). Ten cases reported the date of onset of symptoms was the same as the date of the last sexual contact, and three reported the last sexual contact date was after the onset of symptoms. There were 27 cases where men did not remember the date of the last sexual contact or this information was not recorded in the clinical notes. Figure 3 illustrates the intervals between the last reported sexual contact and the onset of any urethral symptoms. The mean time between the onset of symptoms and presentation to the clinic was 5.6 days (SD 6.0, ranging from 1 to 30 days). Among the 116 cases with urethral gonorrhoea, 112 cases were also tested for urethral chlamydia and 27 (24.1%) tested positive for urethral chlamydia. Of the 27 cases co-infected with urethral gonorrhoea and chlamydia, 20 had typical discharge, five had atypical symptoms and two had no symptoms (Table 2).

## Discussion

We estimated that the positivity for urethral gonorrhoea was 1.3% among heterosexual men attending a sexual health clinic in Melbourne, Australia. Among the 116 confirmed urethral gonorrhoea infections, we found that 80.2% (95% CI: 71.9–86.5%) of cases had typical urethral symptoms and only 6.0% (95% CI: 2.7–12.1%) were asymptomatic on the day of diagnosis, which is consistent with past studies [13, 14].

This study includes only men who attended a sexual health clinic; therefore, the proportion of asymptomatic cases might be underestimated when compared to the general population of heterosexual men, as men without symptoms may not seek healthcare and remain unnoticed in the community. Numerous studies have also estimated that the proportion of men with asymptomatic urethral gonorrhoea could be up to 40% among heterosexual male contacts of females with gonorrhoea [15, 16]. Population-based studies are useful to estimate the proportion of asymptomatic urethral gonorrhoea but also are difficult to conduct given the prevalence of gonorrhoea among heterosexuals is relatively low.

This study shows that the majority of heterosexual men diagnosed with urethral gonorrhoea reported typical urethral discharge at the time of diagnosis. Although in our study the proportion of heterosexual men who were asymptomatic was almost two-fold lower than in MSM (11%), this may relate to the more frequent screening in MSM than heterosexuals and therefore increased likelihood of detecting recently acquired infections before symptoms develop. Priest and colleagues have shown that men with purulent discharge had a higher load of *N. gonorrhoeae* (3.7 × 10^6^ copies per swab) than men with asymptomatic urethral gonorrhoea (2.0 × 10^5^ copies per swab) [17].

We calculated the time between the last reported sexual contact and the onset of symptoms as a proxy for the incubation period, as the exact time of infection and the transmitting partner’ of gonorrhoea is not known. The mean duration in our study was 7 days in heterosexual men which is comparable to past studies [14, 16, 18]. However, a previous study by our team has shown that the time between the last reported sex and onset of symptoms was only 3.9 days among men who have sex with men [13]. We also noted that the time from onset of symptoms to healthcare seeking also seems to be longer among heterosexual men (5.6 days) than we had previously reported for MSM (3 days). It is possible that heterosexual men are more reluctant in seeking healthcare than MSM or have lower levels of knowledge and awareness of STIs [19, 20]. This reduced health literacy among heterosexuals could relate to most of the existing sexual health programs and interventions mainly targeting MSM rather than heterosexuals. With the rise in STI in heterosexuals, these programs should also target the heterosexual population including STI symptoms recognition and awareness.

There are some limitations in the study that should be considered when interpreting these results. First, this study was conducted in one urban major public sexual health clinic, which may not be generalizable to other settings such as general practices. It is possible that men attending sexual health clinics are more likely to be sexually active, engage in healthcare seeking, and be more aware of the STI-related symptoms. Second, recall bias might have occurred when the men reported the time between the onset of symptoms and last sexual contact. Additionally, a substantial proportion of data was missing because this data was not routinely collected, or men could not recall the time of last sexual contact and the onset of symptoms.

## Conclusion

In conclusion, one of every five urethral gonorrhoea cases were among men without typical urethral symptoms, we recommend heterosexual men should be screened for gonorrhoea in the presence of urethral symptoms even if typical discharge is absent.

## Data Availability

All data generated or analysed during this study are included in this published article.

## Abbreviations

AC2: Aptima Combo 2
CI: Confidence intervals
HIV: Human immunodeficiency
MSM: Men who have sex with men
MSHC: Melbourne Sexual Health Centre
NAAT: Nucleic acid amplification test
SPSS: Statistical Package for the Social Sciences
STI: Sexually transmitted infection
WGS: Whole genome sequencing

## References

[1] Kirby Institute (2018). Annual surveillance report 2018 HIV, viral hepatitis and sexually transmissible infections in Australia.

[2] Chow EPF, Grulich AE, Fairley CK (2019). Epidemiology and prevention of sexually transmitted infections in men who have sex with men at risk of HIV. Lancet HIV.

[3] Misson J, Chow EPF, Chen MY, Read TRH, Bradshaw CS, Fairley CK. Trends in gonorrhoea infection and overseas sexual contacts among females in Melbourne, Australia, 2008-2015. Commun Dis Intell. 2018;42. 10.1016/j.jsxm.2018.04.052. 10.33321/cdi.2018.42.22 30626294

[4] Phillips TR, Fairley CK, Chen MY, Bradshaw CS, Chow EPF. Risk factors for urethral gonorrhoea infection among heterosexual males in Melbourne, Australia: 2007-17. Sex Health. 2019;16(5):508–13. 10.1071/SH19027. 10.1071/SH19027 31203836

[5] Savage EJ, Marsh K, Duffell S, Ison CA, Zaman A, Hughes G (2012). Rapid increase in gonorrhoea and syphilis diagnoses in England in 2011. Euro Surveill.

[6] Velicko I, Unemo M (2009). Increase in reported gonorrhoea cases in Sweden, 2001–2008. Euro Surveill.

[7] Rissel C, Badcock PB, Smith AMA, Richters J, De Visser RO, Grulich AE (2014). Heterosexual experience and recent heterosexual encounters among Australian adults: the second Australian study of health and relatio.Nships. Sex Health.

[8] De Visser RO, Smith AMA, Rissel CE, Richters J, Grulich AE (2003). Heterosexual experience and recent heterosexual encounters among a representative sample of adults. Aust N Z J Public Health.

[9] Needleman R, Chow EPF, Towns JM, Cornelisse VJ, Yang TZT, Chen MY (2018). Access to sexual health services after the rapid roll out of the launch of pre-exposure prophylaxis for HIV in Melbourne, Australia: a retrospective cross-sectional analysis. Sex Health.

[10] Dyer C. Doctors warn local authorities about putting sexual health services out to tender. BMJ. 2014;348. 10.1136/bmj.f7715. 10.1136/bmj.f7715 24385552

[11] Foley E, Furegato M, Hughes G, Board C, Hayden V, Prescott T (2017). Inequalities in access to genitourinary medicine clinics in the UK: results from a mystery shopper survey. Sex Transm Infect.

[12] Williamson DA, Chow EPF, Gorrie CL, Seemann T, Ingle DJ, Higgins N (2019). Bridging of *Neisseria gonorrhoeae* lineages across sexual networks in the HIV pre-exposure prophylaxis era. Nat Commun.

[13] Ong JJ, Fethers K, Fairley CK, Chow EPF, Aung E, Chen MY (2017). Asymptomatic and symptomatic urethral gonorrhoea in men who have sex with men attending a sexual health service. Clin Microbiol Infect.

[14] Sherrard J, Barlow D (1996). Gonorrhoea in men: clinical and diagnostic aspects. Genitourin Med.

[15] Handsfield HH, Lipman TO, Harnisch JP, Tronca E, Holmes KK (1974). Asymptomatic gonorrhea in men: diagnosis, natural course, Prevalence and Significance. N Engl J Med.

[16] Landman GS, Gelmi O (1959). Asymptomatic gonorrhea in the male. South Med J.

[17] Priest D, Ong JJ, Chow EPF, Tabrizi S, Phillips S, Bissessor M (2017). Neisseria gonorrhoeae DNA bacterial load in men with symptomatic and asymptomatic gonococcal urethritis. Sex Transm Infect.

[18] Harrison WO, Hooper RR, Wiesner PJ, Campbell AF, Karney WW, Reynolds GH, et al. A trial of minocycline given after exposure to prevent gonorrhea. N Engl J Med. 1979;300(19):1074–8. 10.1056/NEJM197905103001903. 10.1056/NEJM197905103001903 107450

[19] Collyer A, Bourke S, Temple-Smith M (2018). General practitioners’ perspectives on promoting sexual health to young men. Aust J Gen Pract.

[20] Lorimer K, Martin S, McDaid LM (2014). The views of general practitioners and practice nurses towards the barriers and facilitators of proactive, internet-based chlamydia screening for reaching young heterosexual men. BMC Fam Pract.

